# Prevalence and Pattern of Ocular Diseases Among Children Aged 7-14 Years Visiting a Tertiary Care Teaching Hospital in Central India

**DOI:** 10.7759/cureus.66383

**Published:** 2024-08-07

**Authors:** Mihika Dube, Saroj Gupta, Deepayan Sarkar, Bhavana Sharma, Priyanka

**Affiliations:** 1 Ophthalmology, Ram Krishna Dharmarth Foundation (RKDF) Medical College and Hospital, Bhopal, IND; 2 Ophthalmology, All India Institute of Medical Sciences, Bhopal, Bhopal, IND

**Keywords:** central india, refractive error, children, prevalence, ocular morbidity

## Abstract

Purpose: Ocular morbidity in children hinders their overall development. The prevalence and pattern vary amongst countries as well as within a country. Many ocular diseases if diagnosed and treated on time can prevent ocular morbidity in children to a large extent. The aim of this study is to determine the prevalence and pattern of ocular diseases in children (7-14 years) presenting to the All India Institute of Medical Sciences, Bhopal, a tertiary care teaching hospital in central India.

Methods: This is a single-center, hospital-based cross-sectional study conducted from June 2018 to August 2019. A total of 1276 children between 7 and 14 years of age were included. A thorough ocular examination was done and a diagnosis was noted. Statistical analysis was performed using Microsoft Excel, Version 2013 (Microsoft Corp., Redmond, WA) and IBM SPSS Statistics for Windows, Version 23 (IBM Corp., Armonk, NY). A p-value of <0.05 was considered significant.

Results: Out of 1276 children, 505 (39.6%) were of 7-10 years while 771 (60.4%) were of 11-14 years. There were 563 females (44.1%) and 713 males (55.9%). The most common ocular morbidity was a refractive error, (653; 51.1%), it was significantly higher in the age group 11 to 14 years than in children of 7 to 10 years of age (p<0.03). The second most prevalent ocular morbidity was infection/inflammation of the adnexa (18.8%) of which males were affected more than females (p<0.0005). The other morbidities were squint and neuro-ophthalmological-related diseases (8.3%), followed by trauma (3%), congenital diseases (2.6%), amblyopia (2.4%), degenerative diseases (0.7%), neoplastic (< 0.01%), and miscellaneous disorders (1.6%).

Conclusion: The majority of ocular disorders are preventable and treatable. The most common ocular morbidity in our study is refractive error. We recommend school-based screening programs for timely detection and correction of refractive error and to prevent amblyopia.

## Introduction

Ocular morbidity in children affects their overall growth and development, ability to acquire new skills, school performance, social life, and personality [1-5]. The prevalence of blindness in children is about 0.5 to 1.0/1000 globally [6]. There is an estimated 1.4 million blind children globally with about 1 million living in Asia [7]. Approximately 253 million people globally suffer from mild to severe visual impairment [7,8]. A wide variety of ocular diseases can be seen in children, especially those who are underprivileged, suffering from malnutrition, or dwelling in unhygienic localities with a lack of sanitation and safe drinking water. The spectrum of ocular diseases also differs if the child belongs to a developed or developing nation [9-13]. Few ocular diseases in children can lead to permanent blindness or disability, which hinders their growth and development. One more important factor contributing to childhood blindness is the scarcity of medical infrastructure and manpower in rural areas. Out of the 1.5 million children suffering from severe visual impairment, about one million reside in Asia [14]. It is estimated that one child becomes blind every minute [15]. The positive part is that 50% of this blindness is either treatable or preventable or both [16]. Vitamin A deficiency seen in malnourished children is one such cause of preventable blindness in rural and low socioeconomic class people of developing countries like India [17-19]. It is estimated that worldwide, there are 1.5-5 million children with xerophthalmia, of which 350,000 lose their sight [20]. Refractive errors and strabismus are quite common in children and if left uncorrected lead to amblyopia. School screening programs can regularly check for refractive errors and refer affected children for refractive services, thus helping to reduce the burden of amblyopia [21,22]. Children many a time do not even understand that they have low vision and may never complain, this calls for early active identification and treatment to prevent permanent damage. Thus, it is necessary that a detailed analysis be made on the pattern and prevalence of ocular morbidity in children for a particular region for planning and evaluation of preventive and curative services. Very few hospital-based studies are available on childhood ocular morbidity. By studying the pattern and prevalence of various ocular morbidities in a hospital set up we can decide on how to structure a school or population-based screening program. Judicious use of resources and manpower is of utmost importance in our country. Also, the data will help in improving the existing primary eye care facilities consequently reducing the magnitude of ocular morbidity in children and the number of years of blindness ensuing.

## Materials and methods

This is a single-center, hospital-based, cross-sectional study done at a tertiary eye care center in central India. All children in the age group of 7-14 years presenting to the outpatient department of ophthalmology, All India Institute of Medical Sciences (AIIMS), Bhopal between June 2018 and August 2019, were included in the study. Patients referred from the pediatric department and other interdepartmental references were also included. After explaining the study purpose verbally, patients' and guardians' consent was taken in writing in their own language. A total of 1276 children were enrolled in the study. A detailed history was taken both from the children as well as from the parents. All the demographic details such as age, gender, and residential address were noted. A comprehensive ocular examination was done. Visual acuity (VA) was recorded on Snellen's chart at a distance of 6 meters and color vision was checked by using Ishihara's chart. Children with VA<6/9 underwent a pinhole vision to differentiate refractive errors from pathological conditions. Cycloplegic refraction with streak retinoscope was done. Cyclopentolate and homatropine were used for retinoscopy and post mydriatic test was done after three days. Types of refractive errors were classified and noted. Ocular movements were checked uniocularly and binocularly. Convergence weakness was checked. Visual axis alignment was checked using cover-uncover, alternate cover, and Hirschberg tests. Sac was assessed by regurgitation test and syringing was done in selected cases. Slit lamp examination was done for anterior segment pathologies in each and every case. Corneal pathologies such as abrasions, defects, and ulcers were stained with fluorescein. Intraocular pressure measurements by an applanation tonometer were carried out. Fundus examination was done with direct ophthalmoscope, indirect ophthalmoscope, and 90 D slit lamp biomicroscope. Abnormal fundus findings were confirmed on optical coherence tomography. Serological tests were done when required. Orbital and cranial imaging were done in cases of trauma and suspected optic nerve pathologies and intracranial involvement. Children with chronic headaches were consulted by a physician and otolaryngologist as per our discretion. Any other specialist opinion was done as needed. The study was conducted under the aegis of the declaration of Helsinki and permission to conduct the study was taken from the Institutional Ethics Committee of AIIMS, Bhopal (Approval No.: LOP/2018/IM0197).

Inclusion criteria

All children attending the outpatient department of ophthalmology, AIIMS, Bhopal, during the study period and parents giving consent for the study.

Exclusion criteria

Children beyond the age group of the study (below 7 years and above 14 years) and those with no parental consent.

Statistical analysis

Statistical analysis was performed using Microsoft Excel, Version 2013 (Microsoft Corp., Redmond, WA) and IBM SPSS Statistics for Windows, Version 23 (IBM Corp., Armonk, NY). Descriptive statistics were obtained first. Variables were categorized into categorical and continuous variables first. The categorical variables were reported using frequency tables and percentages. The continuous variables were reported using mean and standard deviations. The continuous variables were further categorized into parametric and non-parametric variables using the Shapiro-Wilk test and histograms. Tests of association for categorical variables were performed using Chi-square and Fisher’s exact tests, and tests of association for continuous variables were done using T-tests and Mann-Whitney tests. A p-value of <0.05 was considered significant.

## Results

Of the total of 1276 patients, 505 patients (39.6%) were in the age group 7-10 years while 771 patients (60.4%) were in the age group of 11-14 years. There were 563 females (44.1%) and 713 males (55.9%) in our study (Table 1).

**Table 1:** Age and gender distribution

Age	Male	Female	Total
7-10 years	280	225	505
11-14 years	433	338	771
Total	713	563	1276

The most common ocular morbidity seen was refractive error; there were 653 cases of refractive error (653; 51.1%), of which myopic astigmatism was the most common refractive error detected (264/653 = 40.4%). The maximum cases were seen in children in the 11-14 years age group (409/653 = 62.6%). A significant difference was found between the groups of people 7-10 years of age and people 11-14 years of age, with greater morbidity in the latter group. Males were affected more than females, however the difference was not found to be statistically significant (Table 2).

**Table 2:** Age and gender distribution of refractive error

Refractive error	7-10 years	11-14 years	Total	p-value	Males	Females	Total	p-value
Myopia	77	173	250	0.006	144	106	250	0.05
Hypermetropia	39	52	91	0.05	42	49	91	0.17
Myopic astigmatism	107	157	264	0.17	132	132	264	0.23
Hypermetropic astigmatism	15	24	39	0.88	22	17	39	0.64
Mixed astigmatism	6	3	9	0.06	5	4	9	0.87
Total	244	409	653	0.03	345	308	653	0.288

The second most common ocular morbidity in our study was found to be infection/ inflammation of the adnexa. There were 240 cases (18.8%), of which maximum cases were of allergic conjunctivitis (82/240 = 34.1%), with males significantly affected more than females (53:29), followed by infective conjunctivitis (55/240 = 22.9%) (Table 3). A significant difference was found between the groups of people 7-10 years of age and people 11-14 years of age with a greater number of cases in the second group.

**Table 3:** Age and gender distribution of infection/inflammation of the eye

Infection/inflammation	7-10 years	11-14 years	Total	p-value	Males	Females	Total	p-value
Allergic conjunctivitis	45	72	117	0.39	78	39	117	1
Infective conjunctivitis	29	26	55	0.04	48	7	55	0.0002
Stye	8	8	16	0.46	11	5	16	0.85
Chalazion	2	11	13	0.05	4	9	13	0.004
Blepharitis	5	14	19	0.17	11	8	19	0.39
Preseptal cellulitis	6	2	8	0.04	4	4	8	0.31
Dacryocystitis	2	4	6	0.69	2	4	6	0.08
Keratitis	2	4	6	0.69	2	4	6	0.08
Total	99	141	240	0.06	160	80	240	0.0005

One hundred and seven patients (8.4%) had squint and neuro-ophthalmology-related disorders of which the maximum cases were of convergence weakness (44/107 = 41.1%). The majority were males (66/107 = 61.7%) and in the 11-14 years age group (55.1%) with statistically significant differences in the age groups (Table 4).

**Table 4:** Squint and neuro-ophthalmology disorders

Squint and neuro-ophthalmology	7-10 years	11-14 years	Total	p-value	Males	Females	Total	p-value
Convergence weakness	13	31	44	0.007	28	16	44	0.73
Squint and nystagmus	30	19	49	0.001	30	19	49	0.93
Papilledema	2	7	9	0.15	5	4	9	0.69
Optic atrophy	3	2	5	0.48	3	2	5	0.94
Total	48	59	107	0.008	66	41	107	0.32

The other morbidities diagnosed in our study were trauma (3%), congenital (2.6%), amblyopia (2.4%), degenerative (0.7%), neoplastic (<0.01%), and miscellaneous disorders (1.6%) (Table 5).

**Table 5:** Other ocular morbidities

Diseases	7-10 years	11-14 years	Total	p-value	Male	Female	Total	p-value
Ambylopia								
-Anisometropic	5	13	18	0.14	8	10	18	0.17
-Isometropic	7	3	10	0.01	7	3	10	0.24
-Strabismic	0	3	3	0.14	2	1	3	0.66
Total	12	19	31	0.03	17	14	31	0.39
Trauma	23	15	38	0.19	20	18	38	0.74
Congenital	11	15	26	0.43	13	13	26	1
Cataract	4	4	8	0.28	2	6	8	0.53
Degenerative	4	5	9	0.73	7	2	9	0.09
Neoplastic	1	0	1	0.31	0	1	1	0.31
Miscellaneous	13	7	20	0.37	8	12	20	0.54

## Discussion

In our study, the maximum cases of ocular morbidity were seen in the 11-14 years age group, which is similar to other studies done by Singh et al. and Rao et al. where maximum cases were seen in the 11-15 years and 12-17 years age groups respectively [23,24]. It is probably because the older children are aware of their ocular symptoms. This may also imply neglect of parents towards ocular complaints in younger children.

Males are affected more than females. This is similar to other studies too in India [25]. This is probably because of the higher sex ratio and more concern for the male child in our country.

As consistent with most of the studies, whether hospital-based or community-based, refractive error is the most common ocular morbidity in this age group. The prevalence of refractive error is higher as compared to other studies [26,27]. Although a few studies have even shown greater prevalence than us [24,28,29]. A possible explanation for the higher prevalence of refractive error in our study may be the exclusion of children of 0-7 years of age, as younger the children, diagnosis of refractive error is difficult. The huge prevalence of refractive error is an indicator that the vast majority of children with ocular morbidity can be treated completely with minimally invasive measures. Uncorrected refractive error hinders the overall development and poor academic learning of a child and can lead to amblyopia. Maximum cases (90.3%) of amblyopia in our study were due to uncorrected refractive error (ametropic and anisometropic). Thus, early detection by promotion of school screening programs can lead to timely treatment of refractive error, and help in reducing ocular morbidity.

The second most common ocular morbidity is infection/inflammation of the eye (18.8%), of which the most common is allergic conjunctivitis (34.1%). In other hospital-based studies also inflammation and infections of the eye rank next to refractive error. In a study done by Sinha and Dulani, allergic conjunctivitis was (17.92%) and other infections (14.72%) [26]. In another hospital-based study by Rao et al., the prevalence of allergic conjunctivitis is 8.5% [24]. The high number of these cases may be attributed to a hot and dusty climate as well as a lack of hygiene and proper sanitation. Conjunctivitis has been found to be one of the commonest causes of chronic absenteeism in school-going children, which can be prevented by good hygiene practices.

The third common ocular morbidity is squint and related disorders (8.4%) of which maximum cases were convergence weakness. In a study by Sharma et al. in 2017, squint was seen in 8.2%, which is similar to our study [29]. In a study by Singh et al., convergence insufficiency is found in 2.79% [23].

The prevalence of congenital disorders was 2.6% in our study. In a study by Sinha and Dulani, congenital disorders were found in 12.64% [26]. The usual age group in which the congenital disorders are detected is 0-7 years of age [30]. This is the reason for the low prevalence of congenital disorders in our study as compared to other studies as the population included in our study is more than 7 years of age.

We have a large sample size for a hospital-based study, so our results can be assumed to have a significant impact. Our study had limitations also, particularly as it was a retrospective study design. Patients with incomplete data, children with multiple disabilities, those from lower socioeconomic groups, and those from rural communities who did not attend hospital were likely to be underrepresented, so it may not reflect an actual pattern of ocular morbidity. Our study did not include follow-ups with the patients. 

## Conclusions

Ocular morbidity in children leads to many years of disability. The most common disorders accounting for ocular morbidity are preventable and manageable by simple non-invasive methods. The most common ocular morbidity in our study is refractive error. Thus, timely diagnosis and management lead to the prevention of amblyopia. Our study is unique because there exists a paucity of hospital-based studies on childhood ocular morbidity in our region. Such a large hospital-based study among these age groups has not been done in central India. Information obtained from this study might be useful in strengthening existing primary eye care facilities, consequently reducing the prevalence of childhood blindness and severe visual impairment.

The higher prevalence of refractive errors among older children may be due to their better detection of visual problems, suggesting a lack of awareness among parents to detect them earlier. Visual impairment due to refractive errors is an important public health problem as it affects school performance and impairs children's social and behavioral development. Ongoing school eye screening programs should be strengthened to reduce the prevalence of visual impairment due to refractive errors. Mandatory regular school eye screening programs on a yearly basis should be implemented.
